# Robotic‐assisted simultaneous bilateral total knee arthroplasty was associated with a more favorable perioperative profile than a historical conventional cohort

**DOI:** 10.1002/jeo2.70897

**Published:** 2026-08-26

**Authors:** Eduardo Poblete, Robert Partarrieu, Fernando Martin, Nicolás Piderit, Andrea Marré, Rafael Calvo, David Figueroa

**Affiliations:** ^1^ Faculty of Medicine Clínica Alemana ‐ Universidad del Desarrollo Santiago Chile

**Keywords:** hospital stay, perioperative outcomes, robotic‐assisted total knee arthroplasty, simultaneous bilateral total knee arthroplasty, transfusion

## Abstract

**Purpose:**

To compare perioperative, clinical, and functional outcomes between robotic‐assisted and conventional simultaneous bilateral total knee arthroplasty in a historical comparative cohort.

**Methods:**

We conducted a retrospective historical comparative cohort study of patients undergoing simultaneous bilateral total knee arthroplasty at a single high‐volume arthroplasty centre between March 2013 and July 2025. Patients were stratified into a historical conventional cohort or a computed tomography‐based robotic‐arm‐assisted cohort. Outcomes included post‐operative haematocrit, blood transfusion, tourniquet ischaemia time, length of stay, post‐operative complications, and Forgotten Joint Score‐12 values.

**Results:**

Sixty‐two patients were included: 41 in the robotic‐assisted group and 21 in the conventional group. Baseline preoperative haematocrit was higher in the robotic cohort. Post‐operative haematocrit was higher after robotic‐assisted surgery (31.74 ± 4.81% vs. 26.68 ± 4.41%, *p* < 0.001), whereas haematocrit drop did not differ significantly (*p* = 0.295). The robotic‐assisted group had a lower transfusion requirement (14.6% vs. 76.2%, *p* < 0.001). After multivariable adjustment, conventional surgery remained associated with increased odds of post‐operative transfusion (adjusted odds ratio 20.78, 95% confidence interval 3.19–135.24). Robotic assistance was also associated with shorter tourniquet ischaemia time and hospital stay. Post‐operative complications occurred only in the conventional cohort, although the difference was not statistically significant. Forgotten Joint Score‐12 values were available for 34 of 62 patients (54.8%) at a median follow‐up of 47.9 months and did not differ significantly between groups (81.06 ± 25.76 vs. 74.83 ± 23.86, *p* = 0.486).

**Conclusions:**

Robotic‐assisted simultaneous bilateral total knee arthroplasty was associated with a more favourable perioperative profile than a historical conventional cohort, particularly regarding transfusion requirement, post‐operative haematocrit, tourniquet ischaemia time, and hospital stay. These findings should be interpreted cautiously because of temporal confounding, incomplete functional follow‐up, and limited sample size.

**Level of Evidence:**

Level III.

**Abbreviations**
ASA: American Society of Anesthesiologists
CI: confidence interval
FJS‐12: Forgotten Joint Score‐12
IQR: interquartile range
OR: odds ratio
POD1: post‐operative day 1
SB‐TKA: simultaneous bilateral total knee arthroplasty
TKA: total knee arthroplasty

## INTRODUCTION

Simultaneous bilateral total knee arthroplasty (SB‐TKA) remains controversial because of concerns regarding perioperative morbidity, blood loss, transfusion requirement, and cardiopulmonary complications compared with unilateral or staged procedures [9, 10, 11, 12]. Nevertheless, in appropriately selected patients, SB‐TKA may offer relevant advantages, including a single anaesthetic event, reduced cumulative hospitalisation, lower overall costs, and a consolidated rehabilitation period [9, 10, 11].

A major concern in SB‐TKA is the cumulative perioperative burden associated with performing both procedures during the same surgical session. In particular, post‐operative transfusion requirement and prolonged recovery remain clinically important issues in this setting [9, 10, 11, 12]. Conventional TKA relies on intramedullary femoral instrumentation, which has been associated with increased physiological stress and may contribute to perioperative blood management challenges [2, 4, 7, 9].

Robotic‐assisted total knee arthroplasty has been introduced to improve component positioning, restore limb alignment, and optimise bone resection while avoiding intramedullary alignment guides [1, 8, 13]. Several studies have shown improved radiographic accuracy with robotic techniques [1, 8, 13]. However, evidence regarding their perioperative impact in simultaneous bilateral procedures remains limited. Available bilateral studies are few and include both simultaneous and sequential settings, with inconsistent findings regarding blood loss, transfusion, and early recovery [1, 2, 4, 7, 13].

Therefore, the purpose of this study was to compare perioperative and clinical outcomes, as well as patient‐reported joint awareness assessed using the Forgotten Joint Score‐12 (FJS‐12), between robotic‐assisted and conventional SB‐TKA in a historical comparative cohort. We hypothesised that robotic‐assisted SB‐TKA would be associated with a more favourable perioperative profile, particularly regarding transfusion requirement and early recovery, while FJS‐12 scores would not differ significantly between groups.

## METHODS

### Study design and patient selection

We conducted a retrospective historical comparative cohort study at a single high‐volume arthroplasty centre. We reviewed all patients who underwent SB‐TKA, defined as replacement of both knees during a single anaesthetic session, between March 2013 and July 2025.

Inclusion criteria comprised patients with bilateral tricompartmental knee osteoarthritis who had failed conservative management. Exclusion criteria included revision arthroplasty, inflammatory arthritis, post‐traumatic osteoarthritis, and prior knee infection. The conventional cohort included patients operated on between March 2013 and August 2018, before institutional adoption of robotic‐arm‐assisted technology, whereas the robotic‐assisted cohort included patients operated on between June 2019 and July 2025. All eligible patients were included in the perioperative and clinical outcome analyses. Functional outcomes were assessed after a minimum follow‐up of 12 months; by final functional data closure on 30 July 2026, all included patients had reached this threshold.

### Surgical technique and perioperative management

Procedures were performed by surgeons from the same arthroplasty team. In all cases, both knees were operated on by a single surgeon during the same anaesthetic session. The order of the operated side was not standardised by laterality; instead, the more complex knee was generally addressed first according to surgeon preference and preoperative assessment.

All procedures were performed through a medial parapatellar approach, and all components were cemented. Patellar resurfacing was performed selectively, although it was carried out in the majority of cases according to surgeon preference and intra‐operative assessment.

Robotic‐assisted cases were performed using a computed tomography‐based robotic‐arm‐assisted system. In both cohorts, simultaneous bilateral procedures were performed using the Stryker Triathlon implant system. Posterior‐stabilised or total‐stabilised constructs were selected according to intra‐operative requirements.

A tourniquet was used in most cases, according to surgeon preference. In the majority of cases, the tourniquet remained inflated throughout each knee procedure, beginning with the first side operated on and being deflated after wound closure before starting the contralateral side. Tourniquet pressure was generally 250 mmHg, although it could be adjusted at the request of the anaesthesiology team.

A fully standardised perioperative protocol was not maintained throughout the entire study period. However, all patients received intravenous tranexamic acid according to the institutional protocol: 1 g at the beginning of surgery and a second 1‐g dose intraoperatively. In cases performed with a tourniquet, the second dose was administered at tourniquet release. Post‐operative drains were strictly avoided. Blood transfusion was indicated for a post‐operative haematocrit < 30% in symptomatic patients or < 24% in asymptomatic patients, with customised multidisciplinary thresholds applied for patients with severe cardiovascular or other cardiopulmonary comorbidities.

Post‐operative analgesia was not completely standardised and depended on anesthesiologist and surgeon preference, including patient‐controlled analgesia in some cases and rescue methadone‐based strategies in others. Thromboprophylaxis consisted of enoxaparin 40 mg subcutaneously once daily for 30 days, starting 6 h after surgery.

### Data collection

Collected demographic variables included age, sex, and American Society of Anesthesiologists (ASA) physical status classification. Intra‐operative variables included operative time, tourniquet use, and total tourniquet ischaemia time. Perioperative laboratory data included preoperative haematocrit and post‐operative haematocrit measured on the first post‐operative day (POD1). Post‐operative outcomes included need for blood transfusion, number of transfused units, length of hospital stay, intensive care unit admission, and post‐operative complications.

Post‐operative complications were defined as orthopaedic adverse events, including thromboembolic events, post‐operative infection, reoperation, and persistent symptoms requiring additional evaluation or treatment. Intensive care unit admissions and their underlying medical causes were analysed as separate perioperative outcomes and were not included in the post‐operative‐complication variable. For patients admitted to the intensive care unit, the indication for admission, its relationship to blood transfusion or post‐operative hemodynamic instability, need for vasopressor or ventilatory support, duration of intensive care stay, and subsequent clinical course were verified from the medical records. Body mass index, detailed comorbidity burden, and chronic anticoagulant use were not consistently available throughout the study period and were therefore not included in the adjusted analyses.

### Outcome measures and statistical analysis

The primary outcome was the binary requirement for post‐operative blood transfusion. Secondary outcomes included post‐operative haematocrit, haematocrit drop, operative and tourniquet ischaemia times, hospital length of stay, intensive care unit admission, post‐operative complications, and patient‐reported joint awareness assessed using the FJS‐12.

The FJS‐12 was administered by telephone or e‐mail after a minimum follow‐up of 12 months. The initial functional assessment was conducted in July 2025. Patients without a previously valid FJS‐12 were contacted again on 30 July 2026, after all patients had reached the minimum follow‐up threshold. Follow‐up duration was calculated from the date of surgery to the date of questionnaire completion. Item responses were scored from 0 to 4 and transformed to a 0–100 scale using the formula 100 − (raw score/48 × 100), with higher scores indicating lower joint awareness. Only valid questionnaires were included, and no missing FJS‐12 responses were imputed.

Continuous variables were assessed for normality and reported as mean and standard deviation or median and interquartile range (IQR), as appropriate. Categorical variables were reported as frequencies and percentages. Between‐group comparisons were performed using Welch's t‐test or the Mann–Whitney *U* test for continuous variables and the chi‐square or Fisher's exact test for categorical variables, as appropriate. Analyses were conducted using available‐case data without imputation.

Because all conventional procedures were performed using a tourniquet and only four robotic‐assisted procedures were performed without one, an exploratory analysis of the association between tourniquet use and perioperative outcomes was restricted to the robotic‐assisted cohort. Fisher's exact test was used for transfusion requirement, and the Mann–Whitney *U* test was used for continuous outcomes. This exploratory analysis was not incorporated into the multivariable model because of the small number of procedures performed without a tourniquet.

The association between surgical technique and post‐operative blood transfusion was assessed by calculating crude odds ratios (ORs) with corresponding 95% confidence intervals (CIs). Multivariable logistic regression was performed to evaluate the independent association between surgical technique and post‐operative blood transfusion, adjusting for preoperative haematocrit, age, and ASA classification. The regression analysis was based on complete cases with available data for all model covariates (*n* = 45). Statistical significance was set at *p* < 0.05. No a priori sample size calculation was performed because this retrospective study included the entire available institutional cohort during the study period.

Statistical analyses were performed using Python version 3.13.5, with SciPy version 1.17.0 and statsmodels version 0.14.6.

### Ethical aspects

This retrospective historical comparative cohort study was approved by the institutional ethics committee. Informed consent for the surgical procedure was obtained from all patients, and the requirement for additional study‐specific informed consent was waived by the ethics committee. The study was conducted in accordance with the ethical standards of the 1964 Declaration of Helsinki and its later amendments or comparable ethical standards.

## RESULTS

### Study population

**Figure 1:**
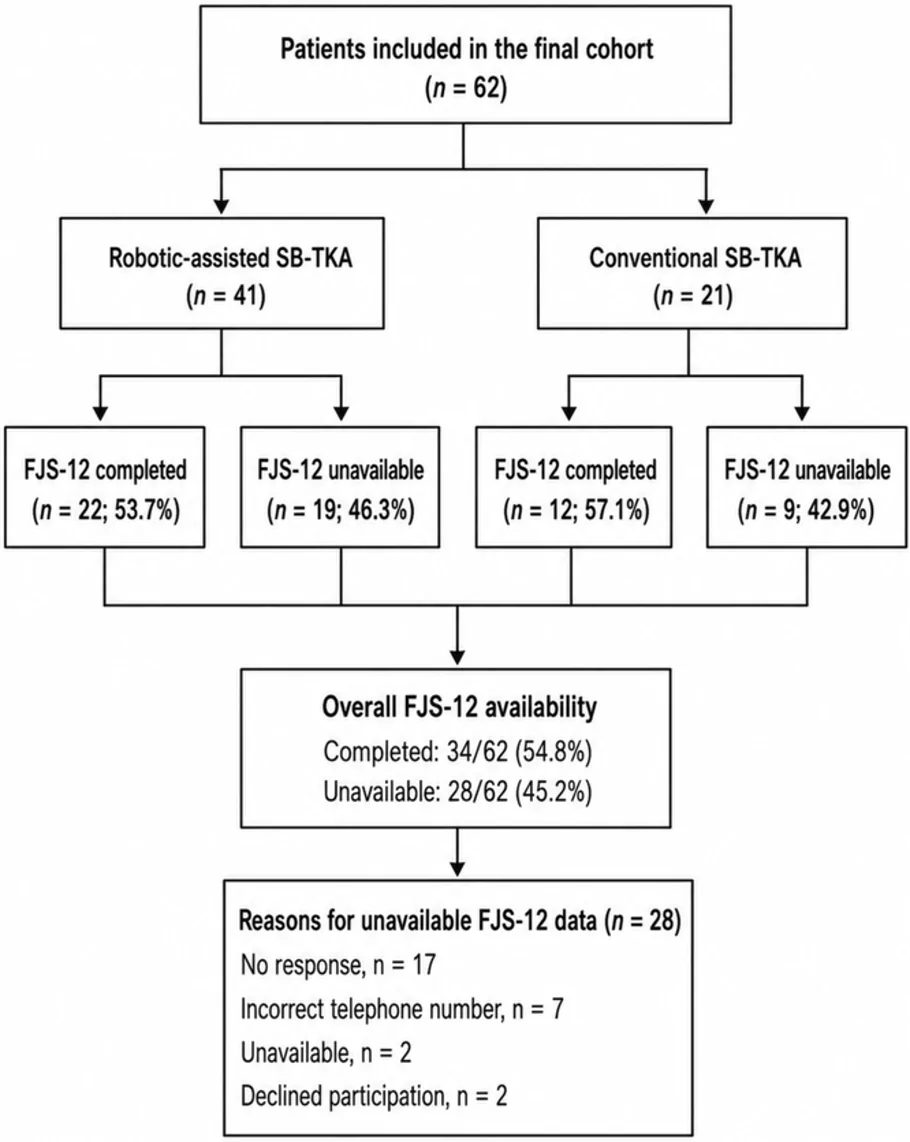
Flow diagram of patient inclusion and availability of functional outcome data. The final cohort comprised 62 patients: 41 in the robotic‐assisted group and 21 in the conventional group. At final data closure, FJS‐12 data were available for 34 patients and unavailable for 28 patients. FJS‐12, Forgotten Joint Score‐12; SB‐TKA, simultaneous bilateral total knee arthroplasty.

A total of 62 patients who underwent SB‐TKA during the study period were included in the perioperative and clinical outcome analyses. Of these, 41 patients underwent robotic‐assisted SB‐TKA and 21 underwent conventional SB‐TKA. Patient inclusion and availability of functional outcome data are shown in Figure 1.

At final functional data closure, all 62 patients had reached a minimum follow‐up of 12 months and were eligible for FJS‐12 assessment. Valid questionnaires were obtained from 34 patients (54.8%): 22 of 41 patients in the robotic‐assisted cohort (53.7%) and 12 of 21 patients in the conventional cohort (57.1%). Functional outcome data were unavailable for 28 patients (45.2%) despite contact attempts, including 19 patients in the robotic‐assisted cohort and nine in the conventional cohort.

### Baseline characteristics

Baseline characteristics are summarised in Table 1. Mean age was comparable between groups (63.56 ± 6.70 years in the robotic‐assisted group vs. 65.41 ± 5.76 years in the conventional group; *p* = 0.265). Sex distribution and ASA physical status classification did not differ significantly between groups.

**Table 1:** Baseline characteristics and intra‐operative parameters.

Variable	Robotic‐assisted SB‐TKA (*n* = 41)	Conventional SB‐TKA (*n* = 21)	*p*‐value
Age, years (mean ± SD)	63.56 ± 6.70	65.41 ± 5.76	0.265
Sex (M/F), *n*	22/19	9/12	0.592
ASA (I/II/III), *n*	10/31/0	4/16/1	0.345
Anaesthesia type, general plus local, *n* (%)	41 (100.0)	21 (100.0)	—
Preoperative haematocrit, % (mean ± SD)	44.36 ± 3.68 (*n* = 32)	40.75 ± 5.25 (*n* = 13)	0.037
Operative time, min (median [IQR])	205 [175–235] (*n* = 41)	190 [170–201.2] (*n* = 8)	0.250
Tourniquet use, *n* (%)	37/41 (90.2)	21/21 (100.0)	0.290
Total tourniquet ischaemia time, min (median [IQR])	127 [112–144] (*n* = 37)	141 [127–161] (*n* = 21)	0.013

*Note*: Analyses were performed using available‐case data. Total tourniquet ischaemia time was analysed only among patients in whom a tourniquet was used.

Abbreviations: ASA, American Society of Anesthesiologists; F, female; IQR, interquartile range; M, male; SB‐TKA, simultaneous bilateral total knee arthroplasty; SD, standard deviation.

Preoperative haematocrit values were available for 45 patients and were significantly higher in the robotic‐assisted group than in the conventional group (44.36 ± 3.68% vs. 40.75 ± 5.25%, respectively; *p* = 0.037).

### Intra‐operative parameters

Intra‐operative parameters are detailed in Table 1. Operative time was available for all 41 robotic‐assisted procedures and for eight conventional procedures. Median operative time did not differ significantly between groups (205 min [IQR 175–235] in the robotic‐assisted group vs. 190 min [IQR 170–201.2] in the conventional group; *p* = 0.250).

Tourniquet use was frequent in both cohorts (90.2% in the robotic‐assisted group and 100.0% in the conventional group; *p* = 0.290). Among procedures performed using a tourniquet, total tourniquet ischaemia time was significantly shorter in the robotic‐assisted group than in the conventional group (127 min [IQR 112–144] vs. 141 min [IQR 127–161], respectively; *p* = 0.013).

In an exploratory analysis restricted to the robotic‐assisted cohort, 37 patients underwent SB‐TKA with a tourniquet and four without a tourniquet. Transfusion occurred in 6 of 37 patients (16.2%) with a tourniquet and in none of the four patients without a tourniquet (*p* = 1.000). No statistically significant differences were detected in post‐operative haematocrit (31.41 ± 4.90% [*n* = 30] vs. 35.03 ± 2.04% [*n* = 3]; *p* = 0.159), haematocrit drop (13.02 ± 4.21% [*n* = 23] vs. 7.95 ± 0.07% [*n* = 2]; *p* = 0.109), length of hospital stay (3.81 ± 1.22 vs. 3.50 ± 0.58 days; *p* = 0.766), or FJS‐12 scores (81.56 ± 25.88 [*n* = 20] vs. 76.04 ± 33.88 [*n* = 2]; *p* = 0.954). Median operative time was 200 min [IQR 170–214] with a tourniquet and 310 min [IQR 268.8–345] without a tourniquet (*p* = 0.003).

### Perioperative blood management outcomes

Perioperative blood management outcomes are shown in Table 2. Post‐operative haematocrit measured on the first post‐operative day was significantly higher following robotic‐assisted SB‐TKA (31.74 ± 4.81% vs. 26.68 ± 4.41%; *p* < 0.001). In contrast, mean haematocrit drop did not differ significantly between groups (12.61 ± 4.27% vs. 14.56 ± 5.50%; *p* = 0.295).

**Table 2:** Perioperative outcomes and functional results.

Outcome	Robotic‐assisted SB‐TKA (*n* = 41)	Conventional SB‐TKA (*n* = 21)	*p*‐value
Post‐operative haematocrit, % (mean ± SD)	31.74 ± 4.81 (*n* = 33)	26.68 ± 4.41 (*n* = 20)	<0.001
Haematocrit drop, % (mean ± SD)	12.61 ± 4.27 (*n* = 25)	14.56 ± 5.50 (*n* = 12)	0.295
Blood transfusion, *n* (%)	6/41 (14.6)	16/21 (76.2)	<0.001
Units transfused (median [IQR])	0 [0–0] (*n* = 41)	1 [1–1](*n* = 21)	<0.001
Length of stay, days (mean ± SD)	3.78 ± 1.17	6.38 ± 2.31	<0.001
Intensive care unit admission, *n* (%)	0/41 (0.0)	2/21 (9.5)	0.111
Any complication, *n* (%)	0/41 (0.0)	2/21 (9.5)	0.111
Reoperation, *n* (%)	0/41 (0.0)	1/21 (4.8)	0.339
Periprosthetic joint infection, *n* (%)	0/41 (0.0)	1/21 (4.8)	0.339
FJS‐12 (0–100), mean ± SD^a^	81.06 ± 25.76 (*n* = 22)	74.83 ± 23.86 (*n* = 12)	0.486
FJS‐12 follow‐up, months (median [IQR])^a^	26.8 [19.9–46.7] (*n* = 22)	106.5 [96.1–119.7] (*n* = 12)	<0.001

*Note*: Intensive care unit admission was analysed separately and was not included in the composite post‐operative‐complication outcome.

Abbreviations: FJS‐12, Forgotten Joint Score‐12; IQR, interquartile range; SB‐TKA, simultaneous bilateral total knee arthroplasty; SD, standard deviation.

^a^ FJS‐12 was available in 34 of 62 eligible patients. Follow‐up was calculated from the date of surgery to the date of questionnaire completion.

The robotic‐assisted cohort had a lower post‐operative blood transfusion requirement than the conventional cohort (14.6% vs. 76.2%; *p* < 0.001). The number of transfused units was also lower in the robotic‐assisted group (median 0 [IQR 0–0] vs. 1 [IQR 1–1]; *p* < 0.001).

The association between surgical technique and transfusion requirement is presented in Table 3. Conventional SB‐TKA was associated with higher odds of post‐operative transfusion (OR 18.67, 95% CI 4.96–70.30; *p* < 0.001). After multivariable adjustment for preoperative haematocrit, age, and ASA classification, conventional SB‐TKA remained associated with increased odds of post‐operative transfusion (adjusted OR 20.78, 95% CI 3.19–135.24; *p* = 0.0015) (Table 4).

**Table 3:** Association between technique and transfusion.

Comparison	Odds ratio	95% CI	*p*‐value
Conventional vs. robotic‐assisted SB‐TKA	18.67	4.96–70.30	<0.001

Abbreviations: CI, confidence interval; OR, odds ratio; SB‐TKA, simultaneous bilateral total knee arthroplasty.

**Table 4:** Multivariable logistic regression analysis for post‐operative blood transfusion.

Variable	Adjusted OR	95% CI	*p*‐value
Conventional vs. robotic‐assisted SB‐TKA	20.78	3.19–135.24	0.0015

*Note*: Model adjusted for preoperative haematocrit, age, and ASA classification. Available‐case analysis (*n* = 45).

Abbreviations: ASA, American Society of Anesthesiologists; CI, confidence interval; OR, odds ratio; SB‐TKA, simultaneous bilateral total knee arthroplasty.

### Length of stay and complications

Length of hospital stay was significantly shorter in the robotic‐assisted group than in the conventional group (3.78 ± 1.17 vs. 6.38 ± 2.31 days; *p* < 0.001).

Admission to the intensive care unit occurred in two patients (9.5%) in the conventional group and in none of the robotic‐assisted cases (*p* = 0.111). One patient developed an allergic reaction during blood transfusion and was admitted to the intensive care unit for monitoring. She did not require endotracheal intubation and subsequently recovered without further complications. The second patient developed severe post‐operative hypotension in the setting of multiple medical comorbidities and required vasopressor support and intensive monitoring. After blood transfusion, the patient evolved favourably and was discharged from the intensive care unit after one day.

Overall post‐operative complications occurred in two patients (9.5%) in the conventional group and in none of the robotic‐assisted group (*p* = 0.111). One patient developed a periprosthetic joint infection requiring reoperation, and one patient reported persistent bilateral knee pain following arthroplasty. Reoperation and periprosthetic joint infection each occurred in one conventional patient (4.8%) and in no robotic‐assisted patients (*p* = 0.339 for both comparisons).

### Functional outcomes

Functional outcomes are presented in Table 2. Among the 34 respondents, median follow‐up from surgery to FJS‐12 completion was 47.9 months [IQR 22.7–96.1]. Median follow‐up was significantly shorter in the robotic‐assisted cohort than in the historical conventional cohort (26.8 months [IQR 19.9–46.7] vs. 106.5 months [IQR 96.1–119.7]; *p* < 0.001).

Mean FJS‐12 did not differ significantly between robotic‐assisted and conventional SB‐TKA (81.06 ± 25.76 vs. 74.83 ± 23.86, respectively; *p* = 0.486).

## DISCUSSION

The most important finding of this study was that robotic‐assisted simultaneous bilateral total knee arthroplasty was associated with a more favourable perioperative profile than the historical conventional cohort, including a lower post‐operative transfusion requirement, higher post‐operative haematocrit, shorter tourniquet ischaemia time, and shorter hospital stay. Conventional SB‐TKA remained independently associated with an increased requirement for post‐operative transfusion after adjustment for preoperative haematocrit, age, and ASA classification. FJS‐12 scores did not differ significantly between groups. Post‐operative complications occurred only in the conventional cohort; however, the difference was not statistically significant and the absolute number of events was small. These findings should be interpreted cautiously because of the historical comparison, potential temporal confounding, incomplete functional follow‐up, and limited sample size.

The perioperative safety of simultaneous bilateral TKA remains controversial. Previous meta‐analytic and large database studies have suggested that bilateral procedures may be associated with higher perioperative morbidity than unilateral or staged surgery, particularly regarding cardiopulmonary complications, transfusion burden, and mortality [9, 10, 11, 12]. Restrepo et al. reported increased odds of pulmonary embolism, cardiac complications, and mortality after simultaneous bilateral TKA in their meta‐analysis [12], while Memtsoudis et al. found higher perioperative morbidity and mortality after bilateral arthroplasty despite a lower comorbidity burden in the bilateral cohort [9]. In this context, efforts to optimise perioperative recovery in SB‐TKA remain clinically relevant.

One of the most striking findings in the present study was the lower post‐operative transfusion requirement in the robotic‐assisted cohort. This observation is clinically important because transfusion remains a relevant concern in bilateral arthroplasty, where the cumulative physiological burden of surgery is amplified. In a prospective randomised bilateral study, Song et al. reported lower post‐operative bleeding on the robotic‐assisted side, although short‐term clinical outcomes were similar between robotic and conventional knees [13]. More recently, Albelooshi et al. demonstrated improved radiographic accuracy with robotic‐assisted simultaneous bilateral TKA, but without clear differences in patient‐reported outcomes at a minimum two‐year follow‐up [1]. Studies evaluating other technology‐assisted bilateral techniques, such as accelerometer‐based navigation, have also shown improved alignment but inconsistent effects on blood loss and transfusion [4, 7]. Jarusriwanna et al. did not demonstrate a reduction in total red blood cell loss or transfusion volume in one‐stage sequential bilateral TKA using accelerometer‐based navigation [4], and Laoruengthana et al. similarly reported improved alignment without a clear reduction in blood loss or transfusion [7].

In the present study, post‐operative haematocrit on the first post‐operative day was higher in the robotic‐assisted cohort, whereas haematocrit drop did not differ significantly between groups. For that reason, our findings are better interpreted as reflecting differences in post‐operative haematologic profile and transfusion requirement rather than definitive evidence of reduced true blood loss. The markedly higher transfusion rate in the historical conventional cohort may have been influenced not only by surgical factors, but also by baseline haematocrit imbalance, individualised transfusion decision‐making, and unmeasured temporal changes in perioperative management. Nevertheless, the persistence of the association after adjustment suggests that surgical technique may still have contributed to the observed difference.

The biological rationale for a more favourable perioperative profile with robotic assistance remains plausible. Conventional TKA commonly relies on intramedullary femoral instrumentation, whereas robotic‐arm‐assisted systems avoid intramedullary alignment guides and may reduce physiological insult related to bone preparation [1, 4, 5, 7, 8, 13]. In bilateral procedures, even modest differences in perioperative stress per knee may become clinically relevant when both knees are replaced during the same anaesthetic session. In addition, robotic systems may improve the reproducibility of bone cuts and soft‐tissue handling. Kayani et al. reported reduced iatrogenic bone and periarticular soft‐tissue trauma in robotic‐arm‐assisted TKA compared with conventional jig‐based TKA [5]. Fontalis et al. subsequently showed that robotic‐arm‐assisted TKA was associated with a lower early local inflammatory response and lower early post‐operative pain, although longer‐term patient‐reported outcomes remained comparable [3]. In parallel, Kayani et al. also reported faster early rehabilitation and shorter time to hospital discharge with robotic‐arm‐assisted unicompartmental knee arthroplasty compared with conventional surgery [6]. Taken together, these studies support the concept that robotic assistance may influence early perioperative recovery even when longer‐term functional outcomes remain similar.

Robotic assistance was also associated with shorter total tourniquet ischaemia time and shorter hospital stay. Because all conventional procedures were performed using a tourniquet and only four robotic‐assisted procedures were performed without one, the independent association between tourniquet use and perioperative outcomes could not be evaluated across the entire cohort. In an exploratory analysis restricted to the robotic‐assisted cohort, no statistically significant differences were detected according to tourniquet use in transfusion requirement, post‐operative haematocrit, haematocrit drop, length of stay, or FJS‐12. Operative time was longer in the four procedures performed without a tourniquet; however, this finding is likely influenced by case selection, changes in surgical workflow, and the very small size of the no‐tourniquet subgroup. Therefore, the exploratory analysis does not permit causal inference regarding the effect of tourniquet use. Similarly, the shorter hospital stay observed in the robotic‐assisted cohort may reflect not only surgical technique but also broader temporal changes in perioperative care, discharge pathways, and enhanced recovery practices over the study period. Nevertheless, previous studies in robotic knee arthroplasty have also suggested advantages in early recovery and hospital discharge, making the observed association biologically and clinically plausible [3, 6].

Post‐operative complications occurred only in the conventional cohort, although the difference was not statistically significant and the absolute number of events was small. One patient developed a periprosthetic joint infection requiring reoperation, and one patient reported persistent bilateral knee pain following arthroplasty. Intensive care unit admissions also occurred only in the conventional cohort. One admission followed an allergic reaction during blood transfusion, whereas the other was related to severe post‐operative hypotension requiring vasopressor support and intensive monitoring. Both patients subsequently recovered favourably. Given the limited number of events and the historical nature of the comparison, these findings should be considered descriptive and should not be interpreted as evidence of a lower complication risk attributable to robotic assistance.

FJS‐12 scores did not differ significantly between groups. This finding is consistent with previous studies showing improved radiographic accuracy or implant positioning with robotic‐assisted TKA without a clear advantage in patient‐reported outcomes [1, 8, 13]. However, the functional comparison in the present study should be interpreted cautiously. FJS‐12 data were available in only 34 of 62 eligible patients, corresponding to a response rate of 54.8%, and the remaining 45.2% may have introduced selection bias. In addition, follow‐up duration was markedly longer in the historical conventional cohort than in the robotic‐assisted cohort. Consequently, the functional results represent assessments obtained at heterogeneous post‐operative time points and should not be interpreted as demonstrating equivalence between techniques or similar longitudinal recovery. Taken together, the available evidence suggests that the potential advantages of robotic assistance may lie predominantly in perioperative recovery and technical precision rather than in clearly superior patient‐reported joint awareness.

This study has several limitations. First, the comparison was historical rather than contemporaneous, introducing substantial risk of temporal confounding related to perioperative management, transfusion practices, discharge criteria, enhanced recovery pathways, surgical experience, and patient selection. Second, the sample size was limited, particularly for complication analyses and the multivariable model, which produced wide confidence intervals. Although the association between conventional technique and transfusion remained statistically significant after adjustment, the precision of the estimate was limited. Third, tourniquet use, post‐operative analgesia, and aspects of perioperative blood management were not fully standardised throughout the study period. Relevant potential confounders, including body mass index, detailed comorbidity burden, and chronic anticoagulant use, were not consistently available and could not be incorporated into the adjusted analysis. Fourth, several variables had missing data and were analysed using available cases without imputation; operative time was available for only eight patients in the conventional cohort. Fifth, FJS‐12 data were available for only 34 of 62 eligible patients, and the substantial nonresponse rate may have introduced selection bias. Functional follow‐up was also markedly longer in the conventional cohort, limiting direct interpretation of the between‐group comparison. Sixth, the exploratory tourniquet analysis included only four robotic‐assisted procedures performed without a tourniquet and was therefore underpowered and vulnerable to selection bias. Finally, the study was not designed to directly quantify true blood loss beyond haematocrit‐based measures and transfusion outcomes.

Despite these limitations, the study also has strengths. It included all eligible patients from the institutional cohort undergoing SB‐TKA during the study period, focused specifically on a clinically demanding bilateral setting, and evaluated outcomes that are highly relevant to perioperative safety. In addition, the use of a historical comparative framework may still provide useful descriptive information regarding how robotic‐assisted SB‐TKA performs after institutional adoption in real‐world practice.

## CONCLUSIONS

Robotic‐assisted simultaneous bilateral total knee arthroplasty was associated with a lower transfusion requirement, higher post‐operative haematocrit, shorter tourniquet ischaemia time, and shorter hospital stay than a historical conventional cohort, while FJS‐12 scores did not differ significantly between groups. These associations should be interpreted cautiously given the historical design, temporal confounding, incomplete functional follow‐up, and limited sample size.

## AUTHOR CONTRIBUTIONS

All authors contributed to the study conception and design. Material preparation, data collection, and analysis were performed by Eduardo Poblete, Robert Partarrieu, Fernando Martin, Nicolás Piderit, and Andrea Marré. The first draft of the manuscript was written by Eduardo Poblete, and all authors commented on previous versions of the manuscript. Rafael Calvo and David Figueroa supervised the study and critically revised the manuscript. All authors read and approved the final manuscript.

## FUNDING INFORMATION

The authors have no funding to report.

## CONFLICT OF INTEREST STATEMENT

The authors declare no conflicts of interest.

## ETHICS STATEMENT

This study was approved by the Ethics Committee of Clínica Alemana de Santiago (approval number 2013‐01) and was performed in accordance with the ethical standards of the 1964 Declaration of Helsinki and its later amendments or comparable ethical standards. Informed consent for the surgical procedure was obtained from all patients, and the requirement for additional study‐specific informed consent was waived by the ethics committee.

## Data Availability

The data sets generated and/or analysed during the current study are available from the corresponding author on reasonable request.
